# Sound Lateralization Test Distinguishes Unimpaired MS Patients from Healthy Controls

**DOI:** 10.1155/2014/462043

**Published:** 2014-07-15

**Authors:** Joshua H. Bacon, Ilya Kister, Tamar E. Bacon, Eliana Pasternak, Yael Strauchler, Joseph Herbert

**Affiliations:** ^1^Department of Psychology, Stern College for Women, Yeshiva University, 215 Lexington Avenue, New York, NY 10016, USA; ^2^NYU Multiple Sclerosis Care Center, Department of Neurology, NYU School of Medicine, 240 E38th Street, New York, NY 10016, USA

## Abstract

There is an urgent need to develop a practical and reliable clinical measure of disease progression in early and mild MS. We hypothesized that a test of sound lateralization, which is exquisitely sensitive to transmission delays in auditory brainstem, could be more useful for detecting processing speed deficits in mildly impaired MS subjects than standard cognitive tasks. *Objective*. To develop a practical test of sound lateralization for the clinic and to compare performance of MS subjects with variable disability and healthy subjects on Sound Lateralization Test (SLT) and two speed-of-processing tasks. *Design*. 42 healthy controls and 90 subjects with clinically definite MS, divided into no, mild, and moderate disability strata, were administered the Symbol Digit Modalities Test (SDMT), and 3-second Paced Auditory Serial Addition Test (PASAT). *Results*. All of the tests showed an overall difference in performance between controls and the three MS groups, but only the SLT measured a significant difference between controls and the no disability group. *Conclusion*. SLT is rapidly applied, technically simple, and superior to standard processing speed tests for discriminating between healthy controls and nondisabled MS subjects. SLT should be investigated as an outcome measure in early-phase trials and for monitoring early disease progression in the clinic.

## 1. Introduction

There are two main reasons to develop tests for quantifying neurologic deficits in minimally or mildly impaired MS patients. The “gold standard” measure of MS disability, Expanded Disability Status Scale (EDSS), is unreliable in the low range [1] and suffers from a number of well-known methodological limitations [2]. Patients with “stable” low EDSS scores often exhibit increasing functional limitations and radiographic disease progression. By the time neurologic deficits become overt, patients may already have reached EDSS score of 3 or more and entered an irreversible phase of the disease, with a relatively fixed time course to moderate-to-severe disability milestones [3]. For clinical decision making it would be most important to have a reliable, objective measure of disease progression in the early phase of MS, when patients may still be within the “window of opportunity” and an intervention could be of greatest benefit [4, 5].

Secondly, there are no accepted clinical outcome measures for monitoring treatment effects of potentially neuroprotective or restorative agents in phase I or II studies. Smaller-scale studies rely on MRI metrics, such as T1 and T2 lesion burden, whose validity as an outcome measure in MS has been called into question [6]. A clinical measure capable of detecting progression of deficits in mild disease could be used to decide which investigational agents should be advanced through the therapeutic pipeline.

As a candidate for a clinical measure sensitive to changes in early disease, we investigated a test of sound lateralization, a measure of speed of processing in the auditory brainstem. We adapted a technically simple, well-established experimental paradigm in which bursts of pure-tone, low-frequency sounds are presented through earphones with variable interaural delay [7]. When two sound bursts of equal intensity are simultaneously presented to both ears through the earphones, the listener perceives a “fused acoustic image” in the middle of the head. A delay between sound bursts, referred to as “interaural time difference” or ITD, is experienced as a shift of the acoustic image toward the ear in which the waveform leads in time [7]. A number of investigators have demonstrated that MS subjects require longer ITDs to lateralize sounds than healthy controls [8–14].

This is not surprising as sound lateralization is exquisitely sensitive to disruptions of auditory brainstem, a frequent locus of demyelination in MS [15].

We hypothesized that a test of sound lateralization, with its “exceptional dependence upon neural timing in the microsecond range” [9], may prove superior for detecting speed-of-processing deficits in MS patients with minimal impairment compared to the commonly used processing speed tests, such as the Paced Auditory Serial Addition Test (PASAT) [16] and the Symbol Digit Modalities Test (SDMT) [17], that measure more global responses on a coarser time scale. The objective of our study was to compare performance of MS subjects with no, mild, and moderate disability and healthy subjects on the sound lateralization task, PASAT, and SDMT.

## 2. Methods

Ninety subjects who fulfilled 2010 Revised McDonald Diagnostic Criteria for relapsing-remitting MS [18] from NYU MS Care Center in New York and 42 healthy controls were recruited for the study. We excluded subjects with active substance abuse; history of traumatic brain injury; hearing difficulties or reliance on hearing aid. Patients who had experienced a relapse within six weeks, as determined by treating clinicians, were excluded. The NYU Institutional Review Board approved the study protocol and all participants provided written informed consent.

The subjects were administered the Sound Lateralization Test (SLT), described in detail below, SDMT, and the 3-second version of the PASAT by a trained research assistant. MS subjects underwent EDSS assessment by a neurostatus-certified clinician at the same visit as the neurocognitive testing. Only patients with EDSS 0–6.5 (bilateral assistance during ambulation) were included in the study. MS patients were divided into three disability strata as follows: no disability (EDSS = 0 and 1); mild disability (EDSS = 1.5 to 3.5), and moderate disability (EDSS = 4 to 6.5). One-way between-subject ANOVAs were conducted for each of the three tests—the SLT, PASAT, and SDMT—to compare overall performance of the four groups on each of the tests. Each ANOVA was followed by Dunnett's test to compare the three MS disability groups to the controls.

## 3. Sound Lateralization Test

Pure 910-Hz tones with durations of 100 ms were generated using Audacity software and delivered through Sony MDR-V6 Dynamic Stereo Headphones. E-Prime software was programmed to control timing and delivery of stimuli and to record responses of the subjects. The reference stimulus consisted of a binaural tone presented simultaneously to each ear, and the test stimulus consisted of binaural tones for which the tone presented to one ear was delayed relative to the other ear. Each trial consisted of two sound bursts: the reference stimulus (ITD = 0), followed after a 500 ms interval by the test stimulus (variable ITD). After hearing the two stimuli, subjects were asked to indicate whether the test stimulus was perceived to the right, left, or same location as the reference stimulus.

ITD from −400 to +400 *μ*s and could be varied in 10 *μ*s steps. ITD the tone was delayed in the right ear relative to the left, and a positive ITD indicates that delay was in the left ear.

ITD for each ear, a series of trials was presented. In the first trial of the series, ITD of +400 *μ*s or −400 *μ*s. At this setting, all subjects were able to correctly localize the test stimulus as being to the right (+400 *μ*s) or left (−400 *μ*s) of the reference stimulus. ITD, with the order determined randomly. ITD in a correctly perceived displacement relative to the reference stimulus, the magnitude of ITD was decreased the trial was administered for that side. ITD was correctly perceived as being to the right of the reference stimulus, the next trial for a displacement to ITD. ITD in this manner on each subsequent, randomly timed ITD for which the subject perceived the test stimulus as being in the same location as the reference. ITD the lower limit for the threshold. On subsequent trials for that side, ITD until the subject again perceived the test stimulus to be correctly displaced relative to the reference. ITD the upper limit for the threshold. ITD computed by taking the average of the upper and lower limits. Once a threshold ITD was established for one side, ITD and all subsequent trials ITD until the upper and lower limits of threshold ITD were similarly determined for the contralateral side.

## 4. Results

Subject characteristics for the controls and the three disability-stratified MS groups are presented in Table 1.

**Figure 1:**
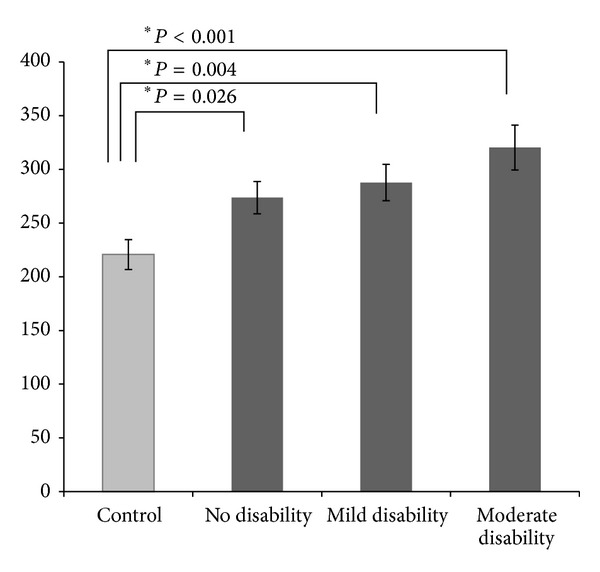
The Mean Interaural Time Difference on the Sound Lateralization Test (in *μ*sec) for the control and three disability-stratified groups.  *Signifies that the difference between the control group and MS groups was statistically significant (*P* < 0.05). Controls did significantly better than each of the MS groups.

There were no significant differences between the left-side and right-side SLT scores for any of the groups. The right and left displacement scores were, therefore, averaged and the mean displacement was used for subsequent analyses. The mean ITD for the controls and the three MS groups are shown in Figure 1. In order to account for the effect of difference in age and gender (see Table 1) on SLT scores, we first conducted a one-way between-subjects ANOVA with group (three disability strata and controls) as the independent variable and age and gender as covariates. We also included as covariates SDMT and PASAT scores to control for differences in level of cognitive impairment. The results showed a significant effect for group (*F*(3,104) = 3.0, *P* = .03; *ηp*2 = 8.0). The effects for all covariates were not significant. With the covariates excluded from the model, a one-way between-subjects ANOVA revealed a highly significant effect for group (*F*(3,110) = 6.4, *P* < 0.001; *ηp*2 = 14.9). Dunnett's test, used to compare the MS groups' means to the control group mean, ITD for the control group (first bar) than for each of the MS disability strata, including the group with normal or near-normal neurologic examination (no disability; second bar).

**Figure 2:**
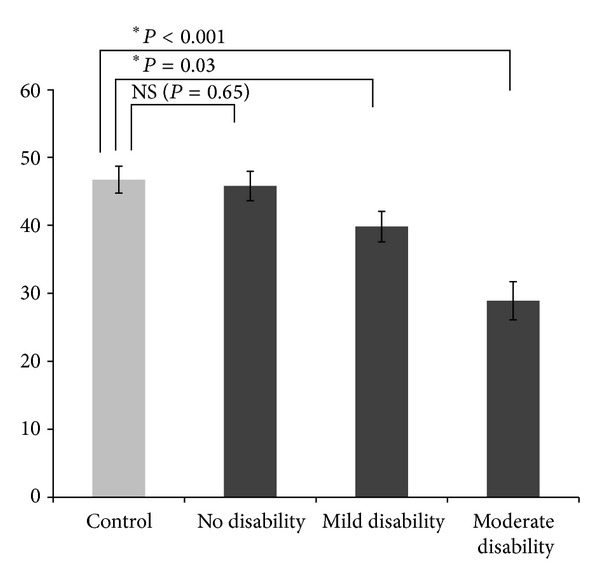
Mean PASAT3 scores for the control and three disability-stratified groups.  *Signifies that the difference between the control group and MS groups was statistically significant (*P* < 0.05).

PASAT results for the control and the MS groups are presented in Figure 2. A one-way between-subjects ANOVA revealed a highly significant effect (*F*(3,126) = 10.40, *P* < 0.001; *ηp*2 = 19.8). Dunnett's test for PASAT scores showed that the PASAT mean for the controls was significantly better than for the moderate disability and mild disability groups but was not significantly different from the no disability group.

**Figure 3:**
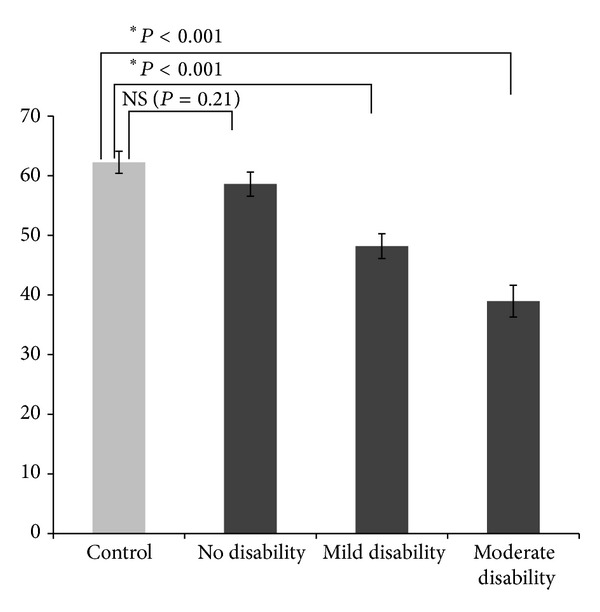
Mean SDMT scores for the control and three disability-stratified groups.  *Signifies that the difference between the control group and MS groups was statistically significant (*P* < 0.05).

Performance of the controls and the MS groups on SDMT is summarized in Figure 3. An ANOVA showed a highly significant effect (*F*(3,126) = 21.4 : *P* < 0.001, *ηp*2 = 33.8) and Dunnett's test showed that the control SDMT mean was significantly better than the moderate disability and mild disability' groups' SDMT means. However, the difference between the control mean and the no disability group was not significant. Thus, while all three tests showed an overall difference in performance between controls and the three MS groups, only the SLT measured a significant difference between the controls and the no disability group.

## 5. Discussion

Our test of sound lateralization measures speed of processing on a time scale that is orders of magnitude smaller than that of standard processing speed tests such as the PASAT and the SDMT. This is due, in part, to the fact that the SLT is primarily a measure of temporal integration, whereas the PASAT and SDMT assess a complex interplay of many cognitive functions—attention, memory, visual or auditory processing, and language. The SDMT and PASAT depend on the integrity of diffuse, higher-level networks [19, 20], while sound lateralization cues are extracted in the auditory brainstem [19, 21]. Thus, impaired sound lateralization has a more straightforward neuroanatomic interpretation [8, 13] as compared to abnormal performance on SDMT or PASAT [19, 21]. For these reasons, we hypothesized that the SLT would be more sensitive to the slowing in speed of processing than the standard tests and sought to develop a simple, practical method of testing sound lateralization in the clinic.

Our study confirms that intra-aural time discrimination is compromised in MS patients as shown by a number of previous investigators [8–14]. The important novel points of our work are, first, that ITD differences between MS subjects and controls were observed across all MS disability strata, including the no disability group (EDSS = 0, 1). Second, SLT was the only test in our battery that showed significant differences between the no disability MS group and healthy controls, while PASAT and SDMT did not. The latter finding supports the hypothesis that SLT is superior for detecting slowed speed of processing in MS than PASAT and SDMT.

Several methodological points deserve mention. In keeping with our goal of designing a brief test procedure, we employed a modified interleaved staircase method to determine threshold ITD. Our results demonstrate that this procedure is successful in discriminating between controls and even the no disability MS group. We are currently evaluating other psychophysical methods that may trade an acceptable increase in testing time for greater sensitivity. Our aim was to develop and test feasibility of a task that would be quick, easy-to-administer, and applicable to the vast majority of MS patients without a history of hearing impairment. We therefore did not require prior assessment for pure-tone hearing loss nor routinely performed full otoscopic and tympanometric examinations. Outside of acute relapses, MS is not associated with significant pure-tone hearing loss [22, 23] and none of our patients had experienced a relapse within six weeks of testing (as per study exclusion criteria). Moreover, none of our patients had known hearing problems and all passed a screening hearing test; they were able to correctly localize sound with the maximum ITD of ±400 ms lateralization.

In summary, we demonstrate that a rapid and technically simple sound lateralization task is superior to PASAT and SDMT for detecting impaired speed of processing in minimally affected MS patients. SLT requires only a personal computer and inexpensive equipment and can be implemented within minutes in a clinic setting. SLT merits further evaluation as a test of choice for quantifying processing speed deficits in MS patients with no or mild neurologic deficits and for early-phase clinical trials. A study assessing longitudinal stability and reproducibility of SLT in mildly affected MS patients is under way.

## Figures and Tables

**Table 1:** Patient characteristics.

	Control group	No disability EDSS 0-1	Mild disability EDSS 1.5–3.5	Moderate disability EDSS 4.0–6.5
Number of subjects	42	35	33	22
Female (%)	62%	67%	73%	82%
Mean age, yrs (SD)	35.4 (10.8)	36.0 (7.8)	41.7 (7.2)	40.4 (7.9)
Mean disease duration, yrs (SD)	NA	6.7 (5.1)	11.9 (7.4)	12.1 (8.0)
Median EDSS	NA	1.0	3.0	6.0
On disease modifying therapy (%)	NA	83%	73%	22%

SD: standard deviation; EDSS: Expanded Disability Status Scale; NA: not applicable.
